# FDG-PET or PET/CT in Fever of Unknown Origin: The Diagnostic Role of Underlying Primary Disease

**DOI:** 10.1155/2011/318051

**Published:** 2011-03-03

**Authors:** Nurhan Ergül, Tevfik Fikret Çermik

**Affiliations:** Clinic of Nuclear Medicine, Istanbul Education and Research Hospital, Samatya, Kocamustafapaşa, Fatih, 34098 Istanbul, Turkey

## Abstract

Fever of unknown origin (FUO) is generally defined as a fever greater than 38.3°C on several occasions during a period longer than 3 weeks for which the etiology behind cannot be diagnosed at the end of at least 1 week hospital stay. Conventional diagnostic methods are still not adequate to reveal underlying reason in approximately 50% of patients with FUO. In patients with certain diagnosis, three major categories are infections, malignancies, and noninfectious inflammatory diseases. Fluoro-18-fluoro-2-deoxy-D-glucose (FDG) is a structural analog of 2-deoxyglucose and accumulates in malignant tissues but also at sites of infection and inflammation. For this reason, FDG PET or PET/CT has great advantage in understanding of underlying pathology in assessment of FUO. However, till today, there are limited studies about the role of FDG PET or PET/CT in evaluation of FUO. In this paper, the impact of FDG PET or PET/CT in the diagnostic work-up of FUO is described by data obtained from literature review.

## 1. Classifications of Fever of Unknown Origin

The classical definition of fever of unknown origin (FUO) was made by Petersdorf and Beeson in 1961 as “a fever that is measured to be above 38.3°C on several occasions during a period longer than 3 weeks for which the etiology behind cannot be diagnosed at the end of at least 1 week hospital stay [1]. In 1991, Durack and Street have made two major changes on this definition. Firstly, they identified and separated FUO types to nasocomial FUO, neutropenic FUO, HIV-associated FUO, that require entirely different clinical approaches in diagnosis and treatment compared with the classical definition. Secondly, the requirement of at least 1-week hospital stay has been replaced with 3-day hospital stay or 3 outpatient visits [2]. The prevalence of FUO among adult-hospitalized patients is reported to be 2.9% [3]. The spectrum of FUO etiology may include more than 200 diseases [2]. The diseases causing FUO vary depending on the geographical differences, development level of countries; and the experience of clinicians [4]. According to the studies conducted to date, the diseases taking part in FUO etiology and their rates are as follows: infections (21–54%), noninfectious inflammatory causes (13–24%), neoplasms (6–31%), and other causes (4–6.5%) [4]. While in Middle East and Far East countries infections are more frequently observed to be the underlying cause of FUO, higher numbers of cases without diagnosis are encountered in developed countries [2]. Tuberculosis (TB) is the most common infectious disease that causes FUO in developing countries; incidence of TB reported in Europe and North America is about 5% [4]. The other commonly encountered infectious diseases are endocarditis, typhoid fever, malaria, brucellosis, cytomegalovirus infection, and AIDS in western countries [4]. Giant cell arteritis, systemic vasculitis, SLE, adult-onset Still's disease, acute rheumatic fever, and polymyalgia rheumatica can be mentioned among noninfectious inflammatory causes [4, 5]. Diseases such as subacute thyroiditis, sarcoidosis, ulcerative colitis, cirrhosis, and drug fever can be mentioned among miscellaneous causes of FUO that are not included in the other groups [4, 6, 7]. 

The FUO causes among geriatric patients do not differ from those in adults [8, 9]. But deep vein thrombosis and temporal arteritis are important considerations in elderly patients [3]. In pediatric patients, infections take the first place among FUO etiologies with a rate of 56.7% and mostly occur as localized infections [10, 11]. As the inflammatory causes constitute 20.9% of all causes, rheumatic fever is the most common one. Neoplasia incidence is 3%, and the rate of undiagnosed cases is around 19.4% [10]. Recently, the number of undiagnosed cases is observed to rise. This may be due to the improvements in diagnostic methods which lead to define the diseases that are hard to diagnose such as FUO [6]. 

## 2. Diagnostic Methods in the Evaluation of Fever of Unknown Origin

In patients presenting with FUO, basic diagnostic methods are performed following detailed history and physical examination. As those methods can differ between the clinics, generally the following are employed: routine biochemical blood tests, complete blood count, peripheral blood film, urinalysis, blood cultures, and chest X-ray [2]. In some centers, abdominal USG and CT along with tuberculin skin test and agglutination tests for Brucella are applied as well [6].

The rate of failure to reach a definitive diagnosis in patients with FUO varies between 7% and 53% [6]. In FUO diagnosis, noninvasive methods are used in 69.2% of cases, whereas invasive methods are preferred in 30.8% of cases [2]. Temporal artery biopsy should be the first-line diagnostic method in absence of diagnostic clues among elderly patients [6, 12]. In cases where imaging modalities are not successful and fine needle aspiration biopsies or excisional biopsies fail, even exploratory laparotomy may be performed. Although laparotomy is a less preferred diagnostic method, its contribution to the diagnostic process has been reported to be 2%–100% in the literature. It can be helpful particularly in TB and hematological malignancies [13]. In selected cases, response to empirical anti-TB therapy may serve as a diagnostic method [8].

## 3. Nuclear Medicine Methods in Determination of Fever of Unknown Origin Etiology

Nuclear medicine methods are generally categorized as secondary diagnostic methods in FUO diagnosis [2, 14]. The most commonly applied methods are Ga-67 citrate scan, labeled leukocyte scan (Tc-99m, In-111), labeled immunoglobulin scan (Tc-99m or In-111), and FDG-PET and PET/CT scan. Ga-67 scan shows acute, chronic, and granulomatous infections and noninfectious inflammation foci. It also demonstrates uptake in malignant tissues, and therefore it could be the first-line nuclear medicine method for investigation of FUO [15, 16]. The assistance of Ga-67 scan in patients with FUO has been reported to be 29% [14]. Its sensitivity and specificity are 67% and 78%, respectively [17]. Low specificity of Ga-67 may be assumed as an advantage considering the wide spectrum of diseases causing FUO [15]. The disadvantages of Ga-67 scan are as follows: low availability, high dose of radiation delivered to the patient, long duration of imaging and reporting, and the risk of superposition of pathological foci on abdomen during intestinal elimination [15, 16]. 

The sensitivity and specificity of In-111-labeled leukocyte scan in detecting the acute and chronic infections are high (60–85%, 78–94%, resp.), and uptake may also be observed in some malignant conditions. Cold areas in scan may reflect the metastatic foci or chronic infections [16, 18]. It has been reported to be superior against Ga-67 scan particularly in revealing the intra-abdominal events [15]. However, In-111-labeled leukocyte scan takes too much time to perform. Moreover, because the photon flux of In-111 is low, it is not suitable for SPECT [16]. Labeled leukocyte scans should be used when there is evidence of a pyogenic focus behind the FUO profile. For instance, occult infection may be mentioned in presence of a history of a surgical procedure in the last 6 months, positive blood cultures, endocarditis, and intravenous or peritoneal catheter, and therefore labeled leukocyte scan may be preferred in these conditions [19].

Labeled immunoglobulin scan is a method that is employed for diagnosing infections and inflammation foci. The advantage of using radiolabeled monoclonal antibodies (mAbs) against surface antigens on granulocytes is an easier labeling procedure not requiring the handling of potentially contaminated blood. However, the high molecular weight, slow diffusion into the inflammation sites, a long plasma half-life, and high uptake in the liver are disadvantages of this method. There are few studies about the use of the commercially available antigranulocyte antibodies. Murine anti-NCA-95 IgG (BW 250/183) labeled with Tc99m recognizes the nonspecific cross-reacting antigen 95 (NCA-95) on human granulocytes. The reported diagnostic sensitivity and specificity for infections were 40% and 92%; PPV and NPV were 88% and 52%, respectively [20]. 

## 4. FDG-PET and PET/CT in Evaluation of Fever of Unknown Origin

Due to wide etiology spectrum of FUO, clinicians still experience difficulties in selecting and applying the diagnostic procedures in those cases. Morphological changes may not occur at early periods of infections and inflammation, both of which constitute the bulk of the FUO etiology. Because of that sensitivities of anatomical imaging, modalities such as USG, CT, and MRI can be low. Moreover, since those modalities only show certain parts of the body, they cannot provide information on pathological events in systemic disorders [21].

Fluoro-18 FDG is a structural analog of 2-deoxyglucose and its half-life is 110 min. There are three transport mechanisms of glucose and F18-FDG into the cells; passive diffusion, active transport by Na^+^-dependent glucose transporter, and the most important one for F18-FDG, with facultative glucose transporters GLUT-1 through GLUT-13. In tumor cells, an increased uptake of F18-FDG is seen; this can be explained by overexpression of the GLUT isotypes—mostly GLUT-1, GLUT-3, and GLUT-5- and overproduction of glycolytic enzymes. The high uptake of F18-FDG in inflammatory cells, granulation tissues, and granulomas is due to the similar mechanisms seen in tumors especially with the overexpression of GLUT-1 and GLUT-3 [22]. FDG-PET is a valuable imaging method for its success in demonstrating both neoplasms and infection-inflammation foci. Recently, FDG-PET scan has been reported to be effective in detection and staging of malignancies and assessment of treatment response. 

Several studies have been reported for the use of FDG-PET or PET/CT in classical FUO patients over the past 2 decades. These studies demonstrated that the contributions to the final diagnosis range from 16% to 89% in patients with FUO by FDG-PET or PET/CT (Table 1).

Similar to Ga-67 scan, the high sensitivity and relatively low specificity of FDG-PET in determining the pathological processes are considered as advantages due to wide spectrum of diseases in FUO etiology [23]. Since the most common 3 etiologies of FUO are known to be infections, noninfectious inflammatory events, and neoplasms, FDG-PET scan appears to be a valuable modality in diagnosing the etiology of FUO. 

The study of Lorenzen et al. [24] is one of the first studies using FDG-PET in diagnosis of FUO, and they found the contribution of FDG-PET to establishment of diagnosis in a group of 16 patients as 69%. They noted the absence of a pathological focus which could be the underlying cause for fever among patients with negative FDG-PET results and reported a high negative predictive value for FDG-PET [24]. Bleeker-Rovers and colleagues [25] retrospectively studied the contribution of FDG-PET to the diagnostic process of patients in whom FUO or suspicious infection and inflammation foci were investigated. While diagnosis could be reached in 46% of 35 patients with FUO, they found the contribution of FDG-PET to the diagnosis as 37%. They reported sensitivity, specificity, positive predictive value, and negative predictive value of FDG-PET as 93%, 90%, 87%, and 95%, respectively [25]. Another study published 2 years later by the same group evaluated the place of FDG-PET in FUO diagnosis by a prospective and systematic approach. This study has been performed before conventional radiological methods and just after basic diagnostic methods. Diagnosis was reached in 50% of 70 patients, and the contribution of FDG-PET to the diagnosis was reported to be 33%. The sensitivity, specificity, positive predictive value, and negative predictive value of FDG-PET were found to be 88%, 77%, 70%, and 92%, respectively. In the same study, abdominal and thoracic CTs were applied on a subgroup of 43 patients. While the positive predictive value of abdominal and thoracic CT was 48% and negative predictive value was 86%, the positive predictive value of FDG-PET was found to be 65% while negative predictive value was 90% [26]. Buysschaert et al. conducted a study on 74 patients with FUO and succeeded to diagnose 53 of them while finding the contribution of FDG-PET as 26% [27]. Jaruskova and Belohlavek reported contribution of FDG-PET or PET/CT to the diagnosis as 36% among 118 (94 had FUO) patients with prolonged fever [21]. Federici et al. performed a study on 14 patients (10 with FUO and 4 with prolonged inflammatory syndrome) and reported the contribution of FDG-PET to the diagnosis as 50% [28]. Keidar et al. [29] prospectively studied the role of FDG PET/CT in 48 patients with FUO. They reported the performance of PET/CT in FUO evaluation showing sensitivity of 100%, specificity of 81%, PPV of 81%, NPV of 100%, and accuracy of 90%. Balink et al. [30] have recently found that the contribution of FDG PET/CT to diagnosis in 68 patients with FUO elevated erythrocyte sedimentation rate and C-reactive protein as 56%. They reported a high negative predictive value of 100% for focal etiologies of FUO excluding the systemic diseases [30]. In one of the most recent studies conducted by Keia et al. [31], FDG-PET/CT was found helpful for diagnosis in 41.6% of 12 patients with FUO. Jasper et al. [32] performed a study about the diagnostic value of FDG-PET and PET/CT in diagnosis of FUO in pediatric patients. In this retrospective study, 47 PET and 30 PET/CT scans were performed in 69 children having unexplained inflammatory signs including fever, increased leucocyte count, CRP, and ESR. Of all the PET and PET/CT scans, 45% were helpful to diagnosis. The final diagnosis was established in 54% of the patients, and among these patients, scans were contributory in 73%. The combination of PET with low-dose CT was found superior to PET without CT in this study [32]. In our unpublished study results, the contribution of FDG-PET/CT on diagnostic process was found to be 50%, and the sensitivity, specificity, positive predictive value, and negative predictive value of FDG-PET/CT were found to be 92%, 45%, 63%, and 100%, respectively. In our study, the underlying etiologies of FUO were neoplasms in 41.6%, infection in 16.7%, noninfectious inflammatory events in 16.7%, and miscellaneous causes in 25% of patients. This result is probably due to elimination of TB and Brucellosis, which are common causes of FUO in our country, by applying specific diagnostic tests at the beginning. Similar to the previous studies in the literature, lymphomas have been found to be the most common reason disease among our patients with neoplasms [4, 33]. 

There are also studies which compare the FDG-PET and other nuclear medicine methods in FUO diagnosis [17, 34, 35]. Meller et al. [17] performed a study on 20 patients with FUO by using a double-head coincidence camera and demonstrated the contribution of FDG-PET to diagnosis as 55%. They applied Ga-67 scintigraphy on a subgroup of 18 patients. The sensitivity, specificity, positive, and negative predictive values of transaxial FDG tomography were found to be 81%, 86%, 92%, and 75%, respectively. The sensitivity, specificity, positive, and negative predictive values of Ga-67 were reported to be 67%, 78%, 75%, and 70%, respectively [17]. Blockmans et al. [34] found the contribution of FDG-PET to the diagnosis of 58 patients with FUO as 41%. They performed both FDG-PET and Ga-67 citrate scan on a subgroup of 40 patients and determined the contribution of FDG-PET to the diagnosis as 35%, while the contribution of Ga-67 scan was 25%. Moreover, entire pathological foci detected on Ga-67 citrate scan were noted to be found on the FDG-PET scan as well [34]. Although Ga-67 scan is preferred as the first-line scanning method due to its success in detection of neoplasms and inflammatory events, its disadvantages such as high radiation dose, long duration of procedure and evaluation, and low spatial resolution render FDG-PET a more valuable method [15–17]. 

The sensitivity and specificity of labeled leukocyte scans in demonstrating infectious and inflammatory events are high. However, because FDG-PET can also display the neoplasms, it seems to be superior in FUO diagnosis. Kjaer et al. [35] compared In-111-labeled leukocyte scan with FDG-PET in 19 patients with FUO and acquired a different result. While they found the sensitivity and specificity of FDG-PET as 50% and 46%, respectively, the sensitivity and specificity of the In-111 granulocyte scan was 71% and 92%, respectively. The positive and negative predictive values of FDG-PET were 30% and 67%, respectively, whereas both of those values were 85% in In-111 scan. Twelve (63%) patients out of 19 were diagnosed; and while infection was determined in 7 patients, autoimmune diseases were detected in 3, uric acid synovitis in 1, and Hodgkin lymphoma in again 1 patient. The FDG-PET was reported to be inadequate for detection of infection foci and its specificity and positive predictive value were found to be low due to false positive results. They reported In-111 as a superior modality for determining infections compared with the FDG-PET [35]. However, although neoplasms constituted only 5% of the etiology spectrum, the rate of neoplasms among etiology of FUO cases has been shown to have the potential to reach up to 30% in the literature [2, 4]. Therefore, since the results obtained were from a specific FUO group, they may not be reflecting the characteristics of overall population. 

FDG-PET has been reported to be superior to other imaging modalities in FUO diagnosis and particularly for detection of vasculitis [17, 28, 34]. Giant cell arteritis (temporal arteritis) and Takayasu arteritis constitute 17% of all FUO causes. FDG uptake has been shown in cases of giant cell arteritis, polymyalgia rheumatica, Takayasu arteritis, peritonitis associated with Wegener granulomatosis, and infectious vasculitis [36]. The sensitivity and specificity of FDG-PET in detection of vasculitis have been reported to be 77–100% and 89–100%, respectively [5]. Mostly, CT and MRI are employed for diagnosis of Takayasu arteritis; however, especially for lesions of early stage, FDG-PET has been found to be more effective [37]. In cases where giant cell arteritis is limited only with temporal arteries, the sensitivity of FDG-PET may be low as a result of small vascular diameter and high background activity in brain [36]. Ferda et al. [38] performed a study with 48 FUO patients using complex FDG PET/CT protocol combined PET and integrated whole diagnostic contrast-enhanced CT with submillimeter spatial resolution. CT data contained diagnostic images reconstructed with soft tissue and high-resolution algorithm. They found the sensitivity of this protocol as 97% and specificity as 75%. In this study, it was emphasized that the whole diagnostic CT investigations play an important role in diagnosis of FUO with assessment of solid abdominal organs, mainly liver parenchyma, intestinal wall, and also the structure of the pulmonary parenchyma where it may be impossible to evaluate the interstitial tissue infiltration with PET because the size of the changes is below its spatial resolution threshold [38].

**Figure 1:**
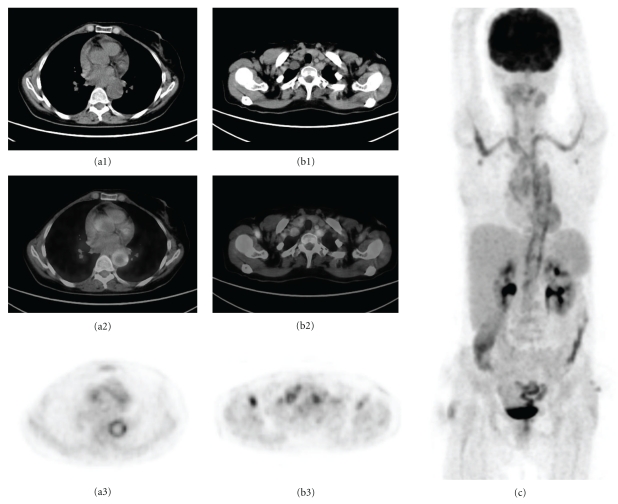
A 54-year-old woman with FUO underwent PET-CT for the diagnosis of underlying disease. Transaxial slices of CT (a1, b1), fusion (a2, b2), and PET (a3, b3) and anterior MIP image (c) showed accumulation of FDG in the wall of thoracic aorta and the supra-aortal branches. Gigantocellular arteritis was confirmed subsequently by temporal arterial biopsy.

**Figure 2:**
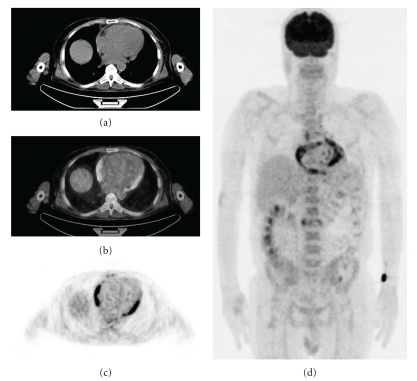
PET-CT of a 63-year-old man with FUO. Transaxial slices of CT (a), fusion (b), PET (c), and anterior MIP image (d) demonstrated increased uptake of FDG in the pericardium. Viral pericarditis was diagnosed by fine needle aspiration biopsy.

**Figure 3:**
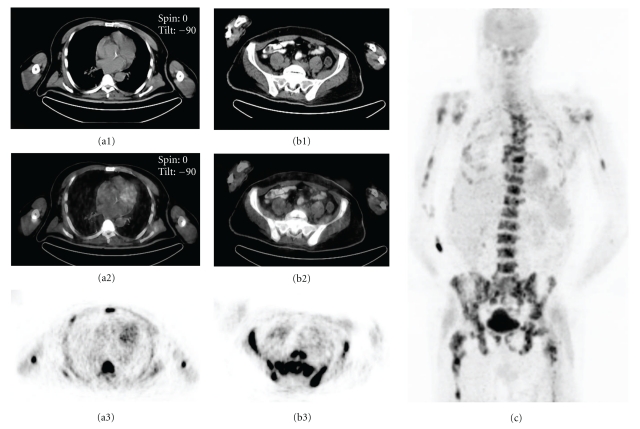
A 58-year-old man with long standing FUO. Diffuse FDG accumulation in bone marrow with no extraosseous manifestations was shown on the transaxial slices of CT (a1, b1), fusion (a2, b2), and PET (a3, b3) and anterior MIP image (c). Non-Hodgkin's lymphoma was diagnosed by bone marrow biopsy.

**Figure 4:**
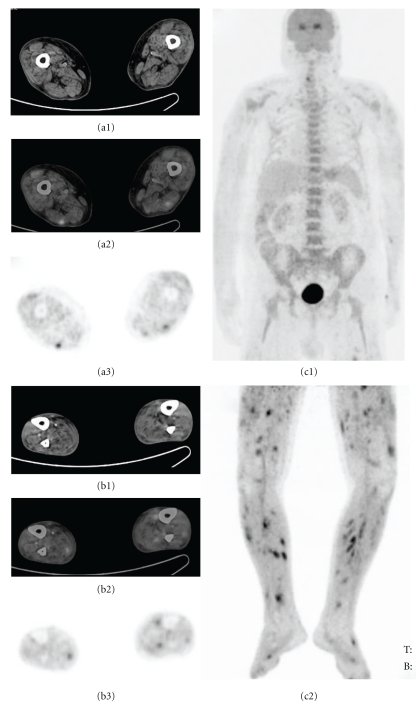
PET-CT of a 77-year-old man with FUO. Transaxial slices of CT (a1, b1), fusion (a2, b2), and PET (a3, b3), and anterior whole body (c1), lower extremities (c2) MIP images revealed multiple foci in the walls of vessels. Nonspecific vasculitis was diagnosed by biopsy.

Examples of diagnostic role of FDG PET/CT in inflammation, infection and neoplasia are presented in Figures 1–4.

## 5. Conclusion and Future Prospects

Although FDG PET/CT is a state-of-the-art procedure for the assessment of multiple malignancies, it is still not a routine procedure in the workup of FUO due to high cost and limited availability. However, the experience with FDG-PET/CT should eliminate application of many unnecessary invasive and noninvasive diagnostic techniques for detection of main disease underlying FUO etiology. Currently, data in the literature indicate that FDG-PET has an important role as a second-line procedure in the management of nearly 50% of patients with FUO. Even though the results of previous FDG-PET studies are promising, still prospective studies using PET/CT on larger populations of FUO are limited. It is well known that hybrid PET/CT improves the diagnostic impact of FDG PET in malignant diseases. For this reason, accurate diagnosis of primary diseases in the context of FUO is expected to increase.

## Figures and Tables

**Table 1:** Review of literature on FDG PET or PET/CT in patients with fever of unknown origin.

Author (Year)	Study design	Patients number	FDG-PET technique	PET helpful (%)	PPV (%)	NPV (%)
Meller et al. (2000)	Prospective	18	Coincidence camera	55	92	75
Blockmans et al. (2001)	Prospective	58	Full-ring PET	41	—	—
Lorenzen et al. (2001)	Retrospective	16	Full-ring PET	69	92	100
Bleeker-Rovers et al. (2004)	Retrospective	35	Full-ring PET	37	87	95
Kjaer et al. (2004)	Prospective	19	Full-ring PET	16	30	67
Buysschaert et al. (2004)	Prospective	74	Full-ring PET	26	—	—
Bleeker-Rowers et al. (2007)	Prospective	70	Full-ring PET	33	70	92
Keidar et al. (2008)	Prospective	48	PET/CT scan	46	81	100
Balink et al. (2009)	Retrospective	68	PET/CT scan	55	93	78
Federici et al. (2010)	Retrospective	10	PET/CT scan	50	—	—
Jasper et al. (2010)	Retrospective	44	Full-ring PET or PET/CT scan	43	—	—
Ferda et al. (2010)	Retrospective	48	PET/CT scan (contrast-enhanced CT)	89	97	75
Keia et al. (2010)	Retrospective	12	PET/CT scan	42	71	100
Ergul et al.*	Retrospective	28	PET/CT scan	50	63	100

	Total:	548	Mean values:	47	78	88

*Unpublished data, PPV: positive predictive value, NPV: negative predictive value.
